# Research Progress on Genomic Regions and Candidate Genes Related to Milk Composition Traits of Dairy Goats Based on Functional Genomics: A Narrative Review

**DOI:** 10.3390/genes15101341

**Published:** 2024-10-19

**Authors:** Xu Yang, Qing Li, Yanyan Wang, Jianmin Wang, Jiaqing Hu, Zhibin Ji, Tianle Chao

**Affiliations:** 1 Shandong Provincial Key Laboratory for Livestock Germplasm Innovation & Utilization, College of Animal Science and Veterinary Medicine, Shandong Agricultural University, Tai’an 271014, China; q1369435187@163.com (X.Y.); l18853852560@163.com (Q.L.); 17826621820@163.com (Y.W.); wangjm@sdau.edu.cn (J.W.); jqh0609@sdau.edu.cn (J.H.); zbji916@sdau.edu.cn (Z.J.); 2 Shandong Provincial Key Laboratory of Animal Biotechnology and Disease Control and Prevention, College of Animal Science and Veterinary Medicine, Shandong Agricultural University, Tai’an 271014, China; 3 Key Laboratory of Efficient Utilization of Non-Grain Feed Resources (Co-Construction by Ministry and Province), Ministry of Agriculture and Rural Affairs, Shandong Agricultural University, Tai’an 271014, China

**Keywords:** goat milk, milk composition, functional genomics, genomics, transcriptomics, proteomics, metabolomics

## Abstract

Background: Goat milk has gained global attention for its unique nutritional properties and potential health benefits. Advancements in functional genomic technologies have significantly progressed genetic research on milk composition traits in dairy goats. Results: This review summarizes various research methodologies applied in this field. Genome-wide association studies (GWAS) have identified genomic regions associated with major milk components, with the diacylglycerol acyltransferase 1 (*DGAT1*) gene and casein gene cluster consistently linked to milk composition traits. Transcriptomics has revealed gene expression patterns in mammary tissue across lactation stages, while the role of non-coding RNAs (such as miRNAs and circRNAs) in regulating milk composition has been confirmed. Proteomic and metabolomic studies have not only helped us gain a more comprehensive understanding of goat milk composition characteristics but have also provided crucial support for the functional validation of genes related to milk components. The integration of multi-omics data has emerged as an effective strategy for elucidating complex regulatory networks from a systems biology perspective. Conclusions: Despite progress, challenges remain, including refining reference genomes, collecting large-scale phenotypic data, and conducting functional validations. Future research should focus on improving reference genomes, expanding study populations, investigating functional milk components, exploring epigenetic regulation and non-coding RNAs, and studying microbiome–host genome interactions. These efforts will inform more precise genomic and marker-assisted selection strategies, advancing genetic improvements in milk composition traits in dairy goats.

## 1. Introduction

Goat milk occupies a significant position in the global dairy market, with its production volume steadily increasing over the past few decades. According to the Food and Agriculture Organization of the United Nations (FAO), global goat’s milk production will reach 19.19 million tons in 2022 [1]. Goat milk has garnered increasing attention worldwide due to its unique nutritional characteristics and potential health benefits. In developing countries and regions, goat milk serves as a important source of nutrition, particularly in areas with limited resources and harsh climatic conditions, where goats can adapt to extreme environments and provide high-quality products [2]. Furthermore, goat milk demonstrates distinct advantages in developed countries, especially in terms of processing technology applications, product diversity, and health benefits [3,4]. Therefore, enhancing the quality of goat milk and optimizing its composition holds substantial practical significance and market value.

Goat milk exhibits significant differences from cow milk in terms of nutritional composition and physical properties, which may have important implications for human digestion and absorption processes. For instance, the average diameter of fat globules in goat milk is smaller, potentially enhancing digestibility [5,6]. Goat milk fat contains a higher proportion of medium-chain fatty acids (caprylic and capric acids) and branched-chain fatty acids, contributing to its distinctive flavor profile [7,8,9]. Moreover, medium-chain saturated fatty acids are more readily digestible and absorbable for infants compared to long-chain saturated fatty acids [10]. The protein composition of goat milk is different from that of cow milk, mainly because the αS1-casein content in cow milk is relatively stable while the αS1-casein content in goat milk is regulated by genetic factors [6]. The concentration of αs1-casein in goat milk is highly dependent on genetic polymorphism, accounting for 25% of total protein in milk from goats with A, B, or C alleles, while goats with O or N alleles do not contain αs1-casein [6,11,12,13]. Consequently, goat milk generally has a lower αs1-casein content, which influences its digestive characteristics. Goat milk contains slightly less lactose than cow milk but has a higher concentration of oligosaccharides [8,14].

Functional genomics is a field of molecular biology that aims to understand the complex relationships between an organism’s genome and its phenotype. In contrast to structural genomics, which focuses on the identification and mapping of genetic sequences, functional genomics seeks to elucidate the functions of genes and their products, as well as the intricate networks of interactions that govern biological processes [15,16]. In the context of dairy science, functional genomics encompasses several key approaches: genome-wide association studies (GWAS), transcriptomics, proteomics, metabolomics, and epigenomics. These methods can be used alone for the screening of markers related to goat milk composition traits and the study of the genetic basis of lactation, or they can be combined in a “multi-omics” manner to provide a holistic view of the biological systems governing milk production and composition [17,18]. These studies are of great significance for the genetic improvement of dairy goats, the enhancement of dairy product quality, and in meeting the diverse needs of consumers.

However, current genetic research on goat milk composition still faces several urgent issues and challenges. For instance, our understanding of the key genes and genetic variations affecting milk composition traits remains insufficient, integrative analytical methods for multi-omics data need further refinement, and applying fundamental research findings to practical breeding practices to achieve precise improvements in milk composition traits is a field awaiting breakthroughs. Therefore, this review aims to systematically summarize and analyze the recent research progress in functional genomics related to goat milk composition traits. Specifically, we will focus on the latest findings in genomics, transcriptomics, proteomics, and metabolomics in revealing the genetic basis of milk composition traits, identify key genes and regulatory networks, discuss the current research shortcomings and challenges, and propose future research directions and suggestions. We hope that this review will provide valuable references for researchers and breeders engaged in goat milk composition genetics, thereby promoting the improvement of goat milk quality and the development of the dairy industry.

## 2. Methods

### 2.1. Literature Search Strategy

We conducted a comprehensive literature search of functional genomics studies on milk composition traits in dairy goats. The search was carried out in the PubMed and Web of Science databases from 1 January 2010 to 31 August 2024. A combination of the following keywords was used: (“goat” OR “Capra hircus”OR “caprine”) AND (“mammary” OR “milk” OR “milk composition” OR “milk component” OR “milk quality”) AND (“functional genomics” OR “functional genomic” OR “genomic” OR “genomics” OR “genome” OR “genome sequencing” OR “GWAS” OR “SNP” OR “CNV” OR “indel” OR “transcriptomics” OR “transcriptomic” OR “transcriptome” OR “non-coding RNA” OR “microRNA” OR “miRNA” OR “long non-coding RNA” OR “lncRNA” OR “circular RNA” OR “circRNA” OR “proteomics” OR “proteomic” OR “proteome” OR “metabolomics” OR “metabolomic” OR “metabolome” OR “lipidomics” OR “lipidomic” OR “lipidome” OR “epigenetic”). Only papers written in English were considered for inclusion. A total of 899 papers were screened.

### 2.2. Inclusion and Exclusion Criteria

**Figure 1:**
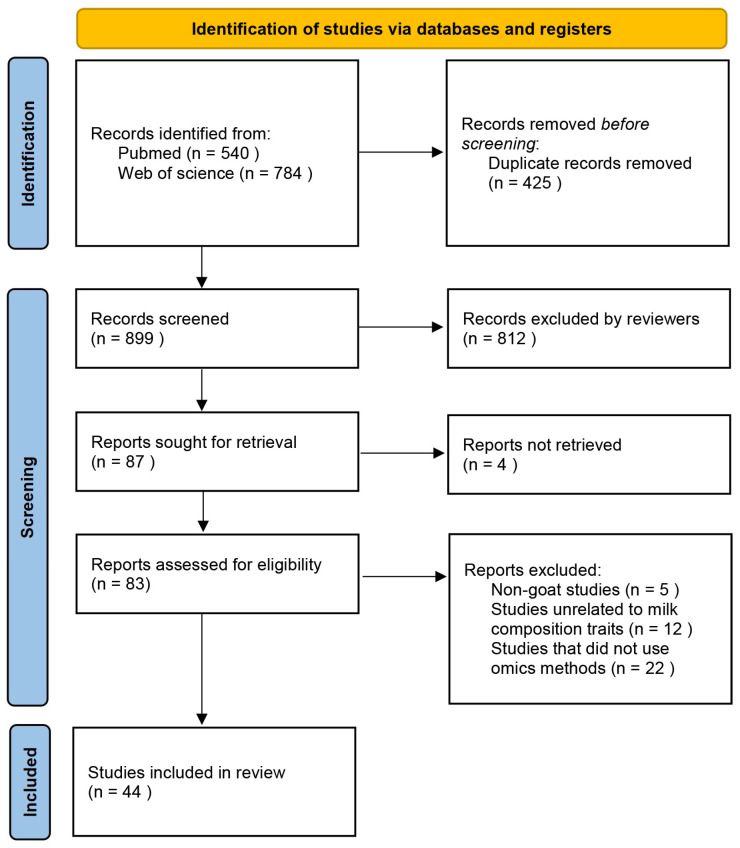
Flow diagram of inclusion and exclusion of papers for this review.

The authors Y. X. and C. T. reviewed the titles and abstracts of the identified articles independently. We assessed articles in more detail using specified inclusion and exclusion criteria. The inclusion criteria are as follows: (1) Focus on functional genomics research related to milk composition traits of goats; (2) the research was carried out by high-throughput omics methods, or the information in the text indicates that the research object molecules are derived from high-throughput omics methods. The exclusion criteria are as follows: (1) Non-goat-related studies; (2) studies unrelated to milk composition traits; (3) studies that did not indicate which specific milk composition traits were studied; (4) studies that did not use omics methods; (5) the full text of the article is not available; (6) reviews, conference abstracts, editorial board articles, and non-peer-reviewed articles. A total of 44 papers were reviewed, analyzed, and summarized for this article (Figure 1).

## 3. Genomics in Goat Milk Composition Trait Research

Genomics, the study of the structure, function, and evolution of an organism’s genome, has provided powerful tools and new perspectives for animal breeding and genetic improvement in livestock production [16]. With technological advancements and cost reductions, the application of genomics in animal husbandry has expanded significantly, progressing from traditional marker-assisted selection to whole-genome selection, greatly enhancing breeding efficiency [19]. In dairy goat research, GWAS and genome-wide selection signal scanning have been used to identify genomic regions and candidate genes associated with economically important traits such as milk yield and composition [20,21].

### 3.1. GWAS Findings: QTLs, Candidate Genes, and Significant SNPs

The fundamental principle of GWAS is to scan a large number of genetic markers (single nucleotide polymorphisms, SNPs) across the entire genome to identify loci significantly associated with target traits [22]. The completion of the goat genome reference sequence and the development of high-density SNP chips have laid the foundation for GWAS research in goats. For instance, the 50K and 52K SNP chips developed by the International Goat Genome Consortium [23,24] and the 66K SNP chip developed by Qiao et al. (2017) [25] have been widely applied in goat genetic studies. The 54K Genome-Wide Liquid SNP Chip for dairy goats developed by Guan et al. (2023) [26] also represents a potentially valuable, cost-effective GWAS tool for milk composition traits. Currently, the majority of GWAS analyses focusing on goat milk composition traits are conducted using high-density SNP chips.

Currently, the GWAS research on the milk composition traits of dairy goats mainly focuses on the main components such as milk fat, milk protein, and lactose. Martin et al. (2017) [21] performed genome-wide association analysis of France goats, including Alpine and Saanen, using a 50K GoatSNP50 Beadchip, and detected 109 regions associated with dairy traits, including 22 regions associated with protein content and 22 regions associated with fat content. The *DGAT1* gene located on chromosome 14 was validated as an important candidate gene for the milk fat content trait, and its exon mutations R251L and R396W were associated with a significant reduction in milk fat content [21].

Guan et al. (2020) [27] conducted a GWAS analysis of milk composition traits in 822 Murciano-Granadina goats using the Goat SNP50 BeadChip and integrated transcriptomic data. They identified three genome-wide significant regions associated with milk composition traits, including QTL1 (chromosome 2, 130.72–131.01 Mb) associated with lactose percentage, QTL6 (chromosome 6, 78.90–93.48 Mb) associated with protein percentage, and QTL17 (chromosome 17, 11.20 Mb) associated with protein and dry matter percentages. Notably, QTL6 overlapped with the location of casein genes [27]. This finding is consistent with Martin et al.’s (2017) [21] GWAS results, which reported significant markers associated with milk protein and fat contents in the vicinity of casein genes [21]. Indeed, numerous studies in cattle have demonstrated that casein genes are the primary functional genes influencing milk protein composition [28,29,30]. Similarly, several studies in dairy goats have reported associations between different genotypes of casein αs1 (*CSN1S1*) and casein kappa (*CSN3*) and milk protein or fat traits [31,32].

Scholtens et al. (2020) [33] conducted a GWAS analysis on 3732 New Zealand dairy goats using the goat 50K SNP chip. They used a combined approach, integrating single-SNP additive linear models (ssGWAS) with three multi-SNP BayesC models. This analysis revealed a significant genomic region on goat chromosome 19 (26.03–26.96 Mb) strongly associated with both fat yield and protein yield. The 15 SNPs within this region accounted for 8.09% of the genomic variance in fat yield and 9.09% of the genomic variance in protein yield. Additionally, they identified a region on chromosome 14 (81.03–81.95 Mb) that explained 2% of the phenotypic variation in fat yield [33].

Luigi-Sierra et al. (2024) [34] conducted genome-wide association analyses for milk composition traits in Murciano-Granadina goats across the first, second, and third lactations using two distinct analytical strategies. Analysis 1 performed independent GWAS for each trait and lactation, while analysis 2 integrated data from all three lactations in a comprehensive GWAS approach. Both analyses identified a QTL associated with lactose percentage on chromosome 2 (129.77–131.01 Mb) and a QTL related to protein percentage in the casein gene cluster region on chromosome 6 (74.8–94.6 Mb). Some QTLs detected in analysis 1 were not consistently observed across all three lactations, suggesting the potential existence of lactation-specific genetic determinants [34].

In contrast to most GWAS studies that focus primarily on major milk composition traits, Selionova et al. (2024) [35] conducted a GWAS analysis on over 20 different milk composition and quality traits in 167 Karachai goats using the goat 53K SNP chip. This comprehensive analysis included detailed fatty acid composition, protein components, lactose, and metabolic indicators such as acetone and β-hydroxybutyrate, which is relatively rare in goat GWAS research. The study identified 40 SNP loci and 65 candidate genes associated with milk composition. Among these, 14 candidate genes were associated with protein and β-casein content, including adrenergic receptor α 1A (*ADRA1A*), NK3 homeobox 1 (*NKX3-1*), NK2 homeobox 6 (*NKX2-6*), stanniocalcin 1 (*STC1*), oocyte-derived antigen 2 (*ODAD2*), tenascin N (*TNN*), Bcl-2-associated agonist of cell death 2 (*BAG2*), neuronal regulator of actin polymerization (*NRAP*), PDZ domain-containing 8 (*PDZD8*), and caspase 7 (*CASP7*). Twenty-five candidate genes were related to fat content and fatty acid composition, such as insulin induction-associated protein 1 (*INSIG1*), PAX interacting protein 1 (*PAXIP1*), bile acid coenzyme A: amino acid N-acyltransferase (*BAAT*), phospholipid phosphatase related 1 (*PLPPR1*), lactamase β (*LACTB*), flavin-containing monooxygenase 1 (*FMO1*), flavin-containing monooxygenase 2 (*FMO2*), enoyl-CoA isomerase 1 (*ECI1*), phosphoglycolate phosphatase (*PGP*), and ATP-binding cassette sub-family A member 3 (*ABCA3).* Seventeen candidate genes were associated with acetone, β-hydroxybutyrate, and urea content, including Ets variant 6 (*ETV6*), activating transcription factor 2 (*ATF2*), neuraminidase 2 (*NEU2*), inositol polyphosphate-5-phosphatase (*INPP5D*), cyclin-dependent kinase 6 (*CDK6*), calcium voltage-gated channel subunit α1 C (*CACNA1C*), sulfotransferase family 2B member 1 (*SULT2B1*), sphingosine kinase 2 (*SPHK2*), fucosyltransferase 1 (*FUT1*), and neurotrophin 5 (*NTN5*). Additionally, genes such as ATP/GTP-binding protein 1 (*AGTPBP1*), NK3 homeobox 1 (*NKX3-1*), NK2 homeobox 6 (*NKX2-6*), and stanniocalcin 1 (*STC1*) were linked to lactose content. Although the study sample size was relatively limited, the research team maximized the available information through exhaustive phenotypic analysis. This in-depth phenotypic characterization lays an important foundation for future larger-scale studies.

In addition to GWAS using SNP chips, recent studies have begun to utilise whole-genome resequencing for milk composition GWAS in goats. For instance, Gudra et al. (2024) [36] utilized whole-genome resequencing data to conduct genetic diversity and GWAS analyses on Latvian local goats, identifying 26 genetic variants associated with somatic cell count, including 18 SNPs and eight insertion/deletion mutations. In GWAS studies of goat milk composition traits, SNP chips remain more prevalent due to their cost-effectiveness, technological maturity, and ease of data processing. However, while less frequently applied, whole-genome resequencing offers unique advantages: it provides higher resolution and comprehensive genomic coverage, enables detection of rare and structural variants, allows direct identification of functional variants, and holds greater potential for novel discoveries [37,38]. In recent years, whole-genome resequencing has become the primary method for detecting genetic variants in GWAS studies of milk composition traits in dairy cattle [39,40,41,42]. Although whole genome resequencing still faces challenges such as high costs, complex data processing, and problems with the quality of the reference genome, its application in goat genetic research is expected to increase with technological advances and cost reductions. This is particularly promising for exploring the precise genetic basis of complex traits.

### 3.2. Genome-Wide Selection Signal Scanning Findings

In addition to GWAS, whole-genome selection signature scans contribute to the detection of functional regions or candidate genes associated with specific milk composition traits. Brito et al. (2017) [20] conducted a selection signature scan on 1151 goats from nine populations, including dairy breeds such as Alpine, Saanen, and Toggenburg, dual-purpose breeds like Rangeland and Nubian, and other production types such as Boer and Cashmere, using the Illumina Goat 50K SNP Beadchip. Their analysis identified selection signals in regions containing genes associated with milk fatty acids (cadherin 12, *CDH12*) and genes related to milk protein and somatic cell count (family with sequence similarity 13 member A, *FAM13A*) [20].

Compared to GWAS, whole-genome selection signature scans lack direct phenotypic associations due to methodological differences, and controlling false-positive risks is more challenging owing to population structure confounding and statistical limitations. Furthermore, selection signatures often involve large genomic regions, and the incomplete genome annotation combined with complex biological pathways makes functional validation more difficult. Moreover, they do not provide quantitative effect estimates. Nevertheless, whole-genome selection signature scans remain a valuable tool for identifying milk composition-related genes. They are particularly useful when combined with other methods, such as GWAS, offering a comprehensive understanding of trait functionality and evolution at the genome-wide level.

## 4. Transcriptomics Studies of Goat Milk Composition Trait Genetics

Transcriptomics studies have provided valuable insights into the molecular mechanisms underlying milk protein synthesis in dairy goats. In candidate gene studies, transcriptome sequencing analysis offers several advantages: it directly reflects gene function, demonstrates strong tissue specificity, captures dynamic changes, and enables the discovery of novel transcripts [18,43]. Moreover, it requires a relatively small sample size. Despite its limitations, such as the lack of genetic variation information, difficulty in establishing causal relationships, and susceptibility to environmental influences, transcriptome sequencing analysis remains a crucial tool for identifying candidate genes associated with goat milk composition.

### 4.1. mRNA Transcriptomics Findings

Messenger RNA (mRNA) transcriptomics plays a pivotal role in elucidating the molecular mechanisms underlying goat milk composition. As the primary mediator of genetic information from DNA to protein, mRNA provides crucial insights into gene expression patterns directly related to milk synthesis and secretion. By examining the expression patterns of multiple genes simultaneously, researchers can identify key metabolic and signaling pathways involved in milk component synthesis.

Currently, transcriptomic studies on the composition of goat’s milk are focusing mainly on elucidating the mechanisms of fatty acid synthesis in milk. Shi et al. (2016) [44] investigated the impact of activating PPARγ, a key gene in milk fatty acid metabolism, on the transcriptome of goat mammary epithelial cells. Their findings suggest that transcription factors may be involved in this process including nuclear receptor subfamily 1 group H member 3 (*NR1H3*), peroxisome proliferator-activated receptor delta (*PPARD*), sterol regulatory element binding transcription factor 1 (*SREBF1*), CCAAT enhancer-binding protein α (*CEBPA*), hypoxia-inducible factor 1 α (*HIF1A*), Jun proto-oncogene (*JUN*), homeobox A9 (*HOXA9*), and cadherin 12 (*CDH12*) [44]. This study elucidates the complex regulatory network governing lipid metabolism in caprine mammary tissue. Li et al. (2020) [45] conducted a comprehensive transcriptome analysis of dairy goat mammary tissue during peak lactation, dry period, and non-pregnant/non-lactating stages. Their research identified several candidate genes potentially regulating milk fatty acid metabolism. These include genes associated with lipid transport and metabolism (phospholipase A2, *PLA2*; carnitine palmitoyltransferase 1, *CPT1*; phospholipase D, *PLD*) and genes involved in intracellular transport, secretion, and vesicular trafficking (Golgi glycoprotein A, *GGA*; signal recognition particle receptor β, *SRPRB*; adaptor protein complex 4 sigma 1, *AP4S1*) [45]. These findings provide insights into the dynamic gene expression patterns across different physiological states of the mammary gland. In a subsequent study, Shi et al. (2022) [46] employed transcriptomic analysis to examine the effects of glucose infusion on goat mammary glands. Their results revealed that glucose infusion promoted de novo fatty acid synthesis in the mammary gland, thereby increasing milk fat yield and elevating the content of caprylic acid and capric acid in milk fat. The treatment also upregulated genes associated with de novo fatty acid synthesis and lipid droplet formation. In vitro experiments corroborated these findings, demonstrating that glucose stimulated the expression of fatty acid synthesis-related genes and cellular triglyceride accumulation in mammary epithelial cells. Mechanistic studies unveiled the crucial role of the AMPK–ChREBP axis in this process. Key genes identified in this pathway include carbohydrate response element binding protein (*ChREBP*), fatty acid synthase (*FASN*), acetyl-CoA carboxylase α (*ACACA*), adipose differentiation-related protein (*ADRP*), butyrophilin subfamily 1 member A1 (*BTN1A1*), elongation of very long chain fatty acids protein 5 (*ELOVL5*), and AMP-activated protein kinase (*AMPK*) [46]. Suárez-Vega et al. (2022) [47] employed RNA-Seq technology to analyze the impact of marine lipid supplementation on gene expression in goat mammary cells, which induced a reduction in milk fat content. Their findings revealed that the downregulation of transcription factors such as sterol regulatory element binding transcription factor 1 (*SREBF1*) and chromatin-modifying enzymes played a crucial role in affecting milk fat synthesis. The study also identified differentially expressed genes, including diazepam binding inhibitor (*DBI*) and glycerol-3-phosphate dehydrogenase 1 (*GPD1),* which formed co-expression networks associated with milk fat depression [47]. This research elucidates the complex regulatory mechanisms involved in the response to dietary lipid supplementation and its effects on milk fat synthesis.

The distinctive flavor profile of goat milk is generally attributed to its unique fatty acid composition. Consequently, transcriptomic studies focusing on fatty acid metabolism in the caprine mammary gland have elucidated several candidate functional genes associated with flavor compounds. Zhang et al. (2024) [48] conducted an integrative analysis combining transcriptomic data from goat, sheep, and cow mammary glands with genomic data from multiple species to elucidate the genetic basis of the unique flavor profile of goat milk. Their study identified methylmalonyl-CoA mutase (*MMUT*), fatty acid synthase (*FASN*), and enoyl-CoA hydratase domain containing 1 (*ECHDC1*) as candidate genes influencing the formation of the characteristic “goaty” flavor in caprine milk. Notably, *FASN* and *MMUT* genes exhibited goat-specific amino acid mutations that may affect the synthesis of branched-chain fatty acids. Furthermore, the research uncovered that the transcription factor zinc finger protein 281 (*ZNF281*) potentially negatively regulates *ECHDC1* expression, which could lead to the accumulation of branched-chain fatty acids, thereby influencing goat milk flavor [48].

While the majority of transcriptomic studies on goat milk have focused on lipid metabolism, a limited number of investigations have also been conducted to elucidate the molecular mechanisms underlying protein and carbohydrate traits in caprine milk. Guan et al. (2020) [27] conducted an integrative analysis combining transcriptome sequencing and GWAS, detecting genes related to milk protein traits (*CSN1S1*; casein β, *CSN2*; casein α S2, *CSN1S2*; *CSN3*), lactose trait (NGFI-A binding protein 1, *NAB1*), dry matter trait (T-box transcription factor 3, *TBX3*), as well as genes potentially associated with calcium ion transport (transient receptor potential cation channel subfamily V member 5, *TRPV5*; transient receptor potential cation channel subfamily V member 6, *TRPV6*) and calcium-phosphorus metabolism (parathyroid hormone-like hormone, *PTHLH;* fibroblast growth factor 23, *FGF23*) [27]. Crisà et al. (2016) [49] performed a comparative transcriptomic analysis of colostrum and mature milk in dairy goats, revealing upregulation of genes related to glycolysis and carbohydrate metabolism in colostrum, particularly those associated with oligosaccharide metabolism [49].

### 4.2. miRNA Transcriptomics Findings

In addition to mRNA studies, research on microRNAs (miRNAs) in goat milk has gained significant attention due to their crucial role in post-transcriptional regulation of gene expression. These small non-coding RNAs, typically 20–24 nucleotides in length, play pivotal roles in various biological processes, including lactation and mammary gland development [50,51]. Recent advancements in high-throughput sequencing technologies have facilitated comprehensive profiling of miRNAs in caprine milk, providing new insights into their potential functions in milk synthesis, composition, and quality [50,51]. Studies on miRNA expression in goat mammary gland have identified several miRNAs that potentially regulate key genes involved in milk fat, protein, and calcium synthesis.

miR-103-5p, discovered by through high-throughput transcriptome sequencing, is a potential molecular breeding target for dairy goats that may regulate the content and composition of goat milk fat, thereby improving milk quality. Lin et al. (2013) [52], by comparing miRNA expression profiles of goat mammary glands during mid-lactation and dry periods, identified miR-103 as highly expressed during lactation. Overexpression of miR-103 in mammary epithelial cells upregulated the expression of genes related to milk fat synthesis (*FASN*; *ACACA*; *SCD*; *DGAT1*; adipose differentiation-related protein, *ADFP;* lipoprotein lipase, *LPL;* solute carrier family 27 member 6, *SLC27A6;* ATP-binding cassette subfamily A member 1, *ABCA1*), leading to lipid droplet formation, triglyceride accumulation, and increased unsaturated fatty acid ratios [53]. Zhu et al. (2024) [54] demonstrated that CRISPR/Cas9-mediated knockout of miR-103 inhibited the accumulation of lipid droplets, triglycerides, and cholesterol in cells. Subsequent knockdown and overexpression experiments proved that miR-103-5p, rather than miR-103-3p, promotes lipid accumulation, and identified phospholipid scramblase 4 (*PLSCR4*) and pantothenate kinase 3 (*PANK3*) as targets of miR-103-5p in this process [54]. Chen et al. (2022) [55] verified in goat mammary epithelial cells that miR-103-5p targets PPARγ to participate in fatty acid metabolism, while circular RNA circ007071 can adsorb miR-103-5p to inhibit lipid accumulation [55].

miR-145 is another miRNA related to fatty acid synthesis in goat mammary glands, discovered through transcriptome studies and experimental validation. Wang et al. (2017) [56], through miRNA expression profile analysis and experimental verification, found that differentially expressed miR-145 promotes lipid accumulation and unsaturated fatty acid synthesis in goat mammary epithelial cells by targeting the Insulin Induced Gene 1 (*INSIG1*) gene. Additionally, inhibition of miR-145 increased DNA methylation levels in the promoter regions of lipid metabolism-related genes such as peroxisome proliferator-activated receptor γ (*PPARG*) and *SREBF1*. Huang et al. (2020) [57] used CRISPR/Cas9 technology to knockout miR-145 in goat mammary epithelial cells, resulting in significantly reduced intracellular triglyceride and cholesterol content, altered fatty acid composition with decreased C16:0 and C20:0 levels and increased C17:0 and C18:0 levels, and downregulation of multiple genes related to fatty acid metabolism, including *SREBF1*, *ACACA*, and *FASN*. Simultaneously, the expression of INSIG1, the target gene of miR-145, was significantly upregulated in knockout cells, and knockdown of *INSIG1* could partially reverse the phenotypic changes caused by miR-145 knockout [57].

Through high-throughput sequencing and functional validation, Chen et al. (2017) [58] uncovered the mechanism by which miR-148a and miR-17-5p synergistically regulate triglyceride synthesis in goat mammary epithelial cells by regulating PPARG coactivator 1 α (*PPARGC1A*) and peroxisome proliferator-activated receptor α (*PPARA*), respectively. The study found that miR-148a and miR-17-5p are highly expressed in lactating goat mammary tissues, targeting *PPARGC1A* and *PPARA*, respectively, to inhibit their expression, thereby promoting triglyceride synthesis and lipid droplet accumulation.

Chen et al. (2016) [59] discovered that miR-30e-5p and miR-15a are highly expressed in goat mammary tissues during peak lactation and synergistically regulate fat metabolism. miR-30e-5p promotes fat metabolism in goat mammary epithelial cells by targeting and inhibiting low-density lipoprotein receptor-related protein 6 (*LRP6*) gene expression, while miR-15a does so by targeting and inhibiting Yes-associated protein 1 (*YAP1*) expression.

Chen et al. (2018) [60], by screening miRNA profiles between peak and late lactation in goat mammary glands, found that miR-181b expression was significantly upregulated during peak lactation. Further studies showed that miR-181b inhibits fat metabolism in mammary epithelial cells by targeting the insulin receptor substrate 2 (*IRS2*) gene and regulating multiple genes in the Hippo signaling pathway [60].

Ma et al. (2018) [61] analyzed miRNA expression patterns in goat mammary tissues at different lactation stages and found that miR-25 expression was lowest during peak lactation. Overexpression of miR-25 significantly inhibited triglyceride synthesis and lipid droplet accumulation in goat mammary epithelial cells. Further research revealed that miR-25 inhibits peroxisome proliferator-activated receptor γ coactivator 1 β (*PGC-1β*) expression by directly targeting its 3‘UTR, thereby affecting the expression of lipid metabolism-related genes such as *SREBP*. These results reveal the important role of miR-25 in lipid metabolism during goat lactation.

Hou et al. (2017) [62] found that miR-574 is differentially expressed between colostrum and peak lactation stages in goat mammary tissues and predicted that it might regulate key genes affecting mammary development and lactation. Zhang et al. (2020) [63] reported that miR-574-5p targets and downregulates ectopic viral integration 5 like (*EVI5L*) to inhibit triacylglycerol and β-casein secretion, and is adsorbed by circRNA-006258. Liu et al. (2020) [64] confirmed that miR-574-5p directly targets and inhibits the expression of mitogen-activated protein kinase kinase kinase 9 (*MAP3K9*) and AKT serine/threonine kinase 3 (*AKT3*), suppressing β-casein and triglyceride secretion, thereby reducing milk synthesis.

Chen et al. (2018) [65], by combining miRNA expression profiles with goat milk fatty acid composition analysis, found that miR-183 expression levels were significantly correlated with various fatty acids, including butyric acid, capric acid, stearic acid, linoleic acid, and arachidic acid. Functional validation in goat mammary epithelial cells showed that miR-183 overexpression significantly reduced cellular triglyceride (TAG) content. miR-183 inhibits milk fat synthesis by targeting and regulating the mammalian Ste20-like 1 (*MST1*) gene. Additionally, miR-183 also affected cholesterol content and β-casein production.

In addition to the aforementioned miRNA transcriptome studies primarily related to lipid metabolism, miRNA transcriptome research associated with goat milk proteins has predominantly focused on caseins. Zhang et al. (2021) [66], combining cell transfection experiments with high-throughput sequencing technology, detected and verified that miR-8516 in goat mammary epithelial cells target and inhibits stanniocalcin 1 (*STC1*) and matrix metalloproteinase 1 (*MMP1*) expression, regulating casein secretion and lipid formation by modulating the phosphorylation of mTOR and STAT5. They further validated the sponge adsorption effect of circRNA circ-140 on chi-miR-8516 [66]. Chen et al. (2018) [67], through transcriptome sequencing and experimental validation, discovered that miR-3031 in goat mammary epithelial cells can activate the PI3K-AKT-mTOR signaling pathway by inhibiting insulin-like growth factor binding protein 5 (*IGFBP5*) expression, thereby promoting β-casein expression. Hou et al. (2024) [68], using high-throughput sequencing, found that miR-2284b is differentially expressed in goat mammary glands during early lactation and peak lactation periods. Their experimental results at the goat mammary epithelial cell level confirmed that miR-2284b targets the 3‘UTR region of the *CSN1S1* gene, negatively regulating *CSN1S1* mRNA expression and αs1-casein production while promoting αs2-casein synthesis.

Furthermore, there have been reports on miRNA transcriptome studies related to calcium components in goat milk. Chen et al. (2020) [69] discovered that miR-99a-3p was differentially expressed in goat mammary glands following 5-hydroxy-l-tryptophan injection, which induced changes in colostrum calcium levels. Experimental results at the mammary epithelial cell level indicated that miR-99a-3p targets and downregulates ATPase sarcoplasmic/endoplasmic reticulum Ca^2+^ transporting 1 (*ATP2B1*), leading to increased intracellular calcium levels. Based on these findings, it is postulated that miR-99a-3p may be involved in the regulation of calcium levels in goat milk.

### 4.3. lncRNA and circRNA Transcriptomics Findings

Non-coding RNAs (ncRNAs) play crucial roles in gene expression regulation and have garnered significant attention in animal science in recent years. In mammary gland biology research, long non-coding RNAs (lncRNAs) and circular RNAs (circRNAs) are believed to potentially participate in the regulatory processes of mammary gland development and milk component synthesis. However, research on ncRNAs in the regulation of goat milk components is still in its infancy, with relatively limited literature available, especially regarding lncRNAs and goat milk component traits.

Yu et al. (2017) [70] conducted sequencing analysis of mRNA, miRNA, and lncRNA in goat mammary tissues during early, peak, and late lactation stages to explore their roles as competing endogenous RNAs (ceRNAs) in the lactation process. Results indicated that upregulated lncRNAs during peak lactation might act as ceRNAs involved in the synthesis and metabolism regulation of lipids, proteins, carbohydrates, and amino acids [70]. Ji et al. (2020) [71], using a similar strategy, performed whole transcriptome sequencing on goat mammary tissues during early, peak, and late lactation stages, identifying differentially expressed lncRNAs related to lipid and protein metabolism. Notably, functional validation experiments on lncRNAs in goat milk component regulation have not yet been reported, highlighting the vast potential for future research in this field.

Circular RNAs (circRNAs) are a special class of non-coding RNA molecules that have received increasing attention in biological research in recent years. As circular molecules, circRNAs are generally considered to have higher stability and resistance to degradation compared to linear RNA molecules. They may influence gene expression related to mammary gland development and milk synthesis by binding to microRNAs (miRNAs) and acting as miRNA sponges. CircRNAs can also participate in forming RNA–protein complexes (RNPs) or regulating RNP activity, or through involvement in epigenetic regulation, and even directly translate into proteins to exert their functions. Current research on the relationship between circRNAs and goat milk component traits primarily focuses on their function as miRNA sponges. The reported circRNA molecules related to goat milk component traits mainly participate in the regulation of milk fat synthesis.

Jiao et al. (2022) [72] identified differentially expressed circRNAs between the dry period and early lactation in dairy goats through whole transcriptome sequencing. They screened and verified that circ003429 regulates triglyceride and unsaturated fatty acid synthesis by binding to miR-199a-3p, thus alleviating the inhibitory effect of miR-199a-3p on the *YAP1* gene. Chen et al. (2022) [55], through high-throughput sequencing and bioinformatics analysis, discovered that circ007071 can regulate *PPARγ* expression by adsorbing miR-103-5p, thereby affecting fatty acid metabolism. Overexpression of circ007071 in goat mammary epithelial cells promoted triglyceride synthesis and increased unsaturated fatty acid content. Wang et al. (2024) [73] investigated the regulatory role of circular RNA circ08436 in lipid metabolism in dairy goat mammary glands. The study found that circ08436 is differentially expressed between dry period and peak lactation goat mammary tissues. Circ08436 can adsorb miR-195 to relieve its inhibition on ELOVL6 gene expression, thereby promoting triglyceride and cholesterol synthesis and increasing saturated fatty acid content. Additionally, m6A methylation modification was involved in the regulation of circRNA-08436, with YTH domain-containing 1 (*YTHDC1*) promoting its transport from the nucleus to the cytoplasm, while YTH domain-containing 2 (*YTHDC2*) recognizes and degrades circRNA-08436 in the cytoplasm [73].

Two studies by Zhang et al. (2020) [63] and Liu et al. (2020) [64] focused on the relationship between circRNAs and both milk fat and milk protein traits. Both studies screened miRNA sponge molecules corresponding to goat miR-574-5p through high-throughput sequencing libraries and verified that circ-016910 and circRNA-006258 in goat mammary epithelial cells can affect β-casein and triglyceride synthesis and secretion by adsorbing miR-574-5p.

## 5. Proteomics of Goat Milk Composition Trait Genetics

Proteomics approaches have provided valuable insights into the protein composition of goat milk and the proteome of mammary tissue, offering a more direct view of the functional molecules involved in lactation, particularly excelling in detecting subtle differences in milk protein components.

By comparing milk proteomes across different mammalian species, conserved functional proteins and species-specific proteins can be identified, aiding in the identification of genes playing crucial roles in goat milk component regulation. Yang et al. (2016) [74] used proteomics methods to construct N-glycosylation protein profiles of milk fat globule membrane (MFGM) fractions from goats, cows, buffaloes, yaks, camels, horses, and humans, providing useful information for further exploration of interspecies differences in MFGM glycoprotein biosynthesis. Yang et al. (2013) [75] analyzed whey proteins from cows, goats, buffaloes, yaks, and camels using iTRAQ quantitative proteomics, revealing higher expression levels of certain proteins in goat milk, including fatty acid synthase, biglycan, C-X-C motif chemokine 6, serum amyloid A protein, and polymeric immunoglobulin receptor fragment. The study also proposed lactose as a potential characteristic marker for goat milk. Anagnostopoulos et al. (2016) [76] analyzed differences in whey proteomes among two goat breeds and three sheep breeds in Greece, finding that β-lactoglobulin content was higher in goat whey than in sheep, particularly in the Capra prisca breed, while CIB1 (calcium-binding protein 1) expression was higher in sheep whey compared to goats. Sun et al. (2019) [77] used proteomics methods to compare whey proteins and milk fat globule membrane proteins between Guanzhong dairy goats and Holstein dairy cows, detecting some unique proteins in goat milk related to diseases and the central nervous system.

By comparing protein expression differences in goat milk from different breeds, lactation stages, or feeding conditions, candidate proteins and genes related to milk component traits can be preliminarily screened. Di et al. (2023) [78] used quantitative proteomics to compare whey proteomes between Teramana and Saanen goat breeds, identifying 29 proteins with significant expression differences between the two breeds. Lu et al. (2016) [79] conducted comparative proteomics analysis of milk fat globule membrane proteins in goat colostrum and mature milk, showing that the abundance of acute phase proteins C3 (complement component 3), LBP (lipopolysaccharide binding protein), FN1 (fibronectin 1), etc., was significantly higher in colostrum MFGM proteins, while the abundance of proteins such as XDH (xanthine dehydrogenase) and STOM (stomatin) increased with the progression of lactation. Sun et al. [80,81] detected and compared milk fat globule membrane proteins and whey proteomes in colostrum and mature milk of Xinong Saanen goats, revealing significant differences in protein composition between colostrum and mature milk. Zhao et al. (2021, 2022) analyzed differential protein expression in Saanen goat milk collected from three different regions (Guangdong, Inner Mongolia, Shaanxi) using quantitative proteomics techniques, identifying heat shock protein 90β family member 1 and haptoglobin as major differentially expressed proteins [82,83].

Proteomics and modified proteomics techniques are also important tools for validating candidate gene functions and elucidating gene action mechanisms. Fu et al. (2021) [84] discovered through nuclear proteomics and RNA-seq analysis that transfection of IGF-I and GH recombinant vectors improved lactation by upregulating PRP1 (proline-rich protein 1), PAG-9 (pregnancy-associated glycoprotein 9), and Hsp70 (heat shock protein 70), and downregulating proteins such as ALB (albumin), TPT1 (tumor protein 1), CXXC-5 (CXXC finger protein 5), and ACTR2 (actin cytoskeleton regulator 2). The IGF-I vector mainly affected purine, pyrimidine, amino acid, and glycerophospholipid metabolism, while the GH vector primarily influenced sphingolipid metabolism, glycerolipid metabolism, retinol metabolism, and the citric acid cycle [84]. Henry et al. (2015) [85] conducted proteomics and phosphoproteomics analyses of goat milk fat globule membrane proteins, revealing a large number of phosphorylated proteins in goat MFGM, with major MFGM proteins such as fatty acid synthase, xanthine dehydrogenase, butyrophilin, and adipophilin exhibiting numerous phosphorylation sites, providing clues for further research on the regulatory role of phosphorylation in mammary gland lipid synthesis and secretion.

## 6. Metabolomics of Goat Milk Composition Trait Genetics

Metabolomics is also an important tool and method for identifying functional genes related to goat milk component traits. Similar to proteomics, metabolomics can detect subtle differences in low-abundance but functionally important components in goat milk. Zhang et al. (2024) [86] conducted a systematic analysis of milk samples from goats, sheep, cows, and buffaloes using widely targeted metabolomics techniques. They identified five core differential metabolites, including 3-(3-hydroxyphenyl)-3-hydroxypropanoic acid, inosine 5‘-triphosphate, methylcysteine, N-cinnamoylglycine, and the dipeptide Tyr-Asn. The study found that goat milk had relatively higher levels of methylmalonic acid, which was predicted to be a candidate molecule for the unique flavor of goat milk. Zhao et al. (2022) [87] performed a comparative analysis of lipid composition in goat, human, cow, sheep, and camel milk, revealing that goat milk contained higher levels of hexosylceramides (HexCer) and ceramides (Cer) compared to other species. Although these metabolomic studies on goat milk components may not directly pinpoint related functional genes or genetic regions, the molecules they identify provide fundamental information for screening genes and genetic markers involved in goat milk component regulation.

Metabolomics not only allows for comprehensive analysis of milk components but can also be integrated with other analytical methods or omics data to reveal relationships between genes, metabolites, and traits from multiple perspectives, providing important scientific basis for milk quality improvement and goat breeding. Caboni et al. (2016) used gas chromatography–mass spectrometry (GC–MS) techniques and multivariate statistical analysis methods to study the metabolomic characteristics of goat milk with different αS1-casein genotypes. The results showed that milk from goats with weak/null alleles contained higher levels of lactose metabolism-related substances, while milk from goats with strong alleles had higher levels of tricarboxylic acid cycle-related substances, revealing changes in milk composition and metabolic characteristics associated with different genotypes [88].

## 7. Future Perspectives and Challenges

With the rapid development of genomics, transcriptomics, proteomics, and metabolomics technologies, significant progress has been made in the genetic research of dairy goat milk component traits. However, this field still faces numerous challenges while harboring immense potential for development.

### 7.1. Technological and Methodological Advancements

As sequencing costs continue to decrease and quality improves, the application of whole-genome resequencing in dairy goat milk component trait research is expected to increase. This will facilitate the discovery of more functional variants, particularly rare and structural variations. Future research should focus more on integrating genomic, transcriptomic, proteomic, and metabolomic data to comprehensively elucidate the regulatory networks of milk component traits. This multidimensional analytical approach will help clarify the complex relationships between genes, proteins, metabolites, and phenotypes. The application of single-cell RNA sequencing technology will contribute to a deeper understanding of gene expression patterns in different cell types within mammary tissue, revealing cell-specific mechanisms of milk component synthesis and secretion. Furthermore, future studies should strengthen research on the role of epigenetic modifications, such as DNA methylation and histone modifications, in milk component regulation, providing new perspectives for understanding the genetic control of milk component traits.

### 7.2. Expansion of Research Focus

Based on current research reports, in addition to existing miRNA and circRNA studies, future research on non-coding RNA functions should strengthen the exploration of other types of non-coding RNAs, such as long non-coding RNAs (lncRNAs), in milk component regulation. Existing studies have mainly focused on major components like milk fat, protein, and lactose. Future research should strengthen genetic studies on functional milk components such as immunoglobulins, lactoferrin, and oligosaccharides, providing a theoretical basis for improving the nutritional value and health benefits of goat milk. Additionally, attention should be paid to gene–environment interactions, with in-depth studies on the interactions between environmental factors (such as feeding management and climatic conditions) and genotypes, contributing to the development of more precise breeding strategies.

### 7.3. Challenges

The collection of large-scale, high-quality, standardized phenotypic data remains a challenge, especially for some difficult-to-measure trace or functional components. Functional validation is also a major hurdle in the practical application of candidate genes and marker loci. Candidate genes or variant sites screened from massive omics data require functional validation, but conducting large-scale functional validation experiments in goats still faces technical challenges. In terms of population size and statistical power, compared to dairy cattle research, dairy goat research populations are usually smaller, which may affect the power of statistical analyses, especially for some polygenic traits with small effects.

Moreover, significant genetic differences exist between different goat breeds, requiring researchers to be cautious when generalizing research results and to consider breed specificity.

### 7.4. Application Prospects

As research continues to deepen, the application of genomic selection in dairy goat breeding is expected to further improve, especially for traits that are difficult to measure or have low heritability. Based on the results of functional genomics research, more precise molecular marker-assisted selection strategies can be developed to enhance breeding efficiency. Additionally, gene editing technologies like CRISPR/Cas9 have broad application prospects in dairy goat breeding, but ethical and regulatory issues still need to be addressed. Personalized nutrition through genotype-based feeding management strategies has the potential to improve the efficiency of dairy goat production and increase milk quality.

## 8. Conclusions

In conclusion, this review highlights the significant advancements in the genetic research of dairy goat milk composition traits through the application of functional genomics. We have summarized various methodologies, including GWAS, transcriptomics, proteomics, and metabolomics, which have collectively enhanced our understanding of the genetic basis underlying milk composition. Key findings indicate that specific genomic regions and candidate genes, such as the *DGAT1* gene and casein gene cluster, play crucial roles in determining milk traits. Despite the progress made, challenges remain in refining reference genomes, collecting large-scale phenotypic data, and conducting functional validations. Future research should focus on integrating multi-omics data to elucidate complex regulatory networks and exploring the roles of epigenetic modifications and non-coding RNAs in milk composition regulation. Additionally, expanding the research scope to include functional milk components and gene–environment interactions will provide a more comprehensive understanding of dairy goat genetics. Ultimately, these efforts will inform more precise genomic and marker-assisted selection strategies, advancing genetic improvements in milk composition traits in dairy goats. By fostering collaboration between academia and industry, we can enhance the quality of goat milk and contribute to the sustainable development of the dairy goat industry.

## Data Availability

No new data were created or analyzed in this study.
